# Editorial: Exploring immunometabolism: metabolic pathway and immune response in sepsis

**DOI:** 10.3389/fmed.2025.1758108

**Published:** 2025-12-17

**Authors:** Yuan Yuan, Lulong Bo, Ruoxi Zhang, Feng Chen

**Affiliations:** 1 Department of Anesthesiology, Shanghai General Hospital, Shanghai Jiao Tong University, Shanghai, China; 2 Department of Anesthesiology, Changhai Hospital, Naval Medical University, Shanghai, China; 3 Center for DAMP Biology, Department of Surgery, University of Texas Southwestern Medical Center, Dallas, TX, United States; 4 Department of Anesthesiology, Ruijin Hospital, Shanghai Jiao Tong University School of Medicine, Shanghai, China

**Keywords:** editorial, immune response, immunometabolism, metabolic pathway, sepsis

Sepsis, defined as a life-threatening systemic inflammatory response syndrome caused by infection, remains a predominant cause of mortality in intensive care units (ICUs) globally, accounting for an estimated 49 million new cases and 11 million deaths annually (Van Dender et al.). Central to its pathogenesis are immunosuppression and metabolic dysregulation, which drive organ dysfunction. This Research Topic, entitled “*Exploring Immunometabolism: Metabolic Pathways and Immune Responses in Sepsis*,” brings together nine articles that comprehensively dissect the molecular mechanisms of immunometabolism in sepsis, identify novel biomarkers, and explore immunometabolism-targeted therapeutic strategies, providing a new theoretical basis for the diagnosis and treatment of sepsis.

A hallmark of sepsis is immune dysfunction, characterized initially by a hyper-inflammatory response followed by persistent immunosuppression. Wang W. et al. systematically reviewed the role and mechanisms of Toll-like receptor (TLR) signaling in sepsis and its multiple organ-related complications, as well as the critical functions of TLRs in the diagnosis and treatment of sepsis and its associated complications. TLRs, as key pattern recognition receptors in innate immunity, are categorized into cell-surface and endosomal types. Each TLR subtype activates distinct downstream signals, triggering specific immune responses. Understanding the regulatory mechanisms of TLR pathways is crucial for developing targeted interventions against immune-related disorders.

Pulmonary involvement is common in sepsis. Alveolar epithelial cells (AECs) are vital for maintaining lung function and actively participate in inflammatory responses. Sepsis-induced damage to AECs—through oxidative stress, microcirculatory disturbances, and inflammation—leads to barrier dysfunction, edema, and respiratory failure. Jia et al. explored AEC pathology in bacterial sepsis-associated ALI, highlighting the role of nuclear factor kappa B (NF-kB), nuclear factor erythroid 2-related factor 2 (NRF2), nucleotide-binding oligomerization domain-like receptor family pyrin domain-containing 3 (NLRP3), and TLR pathways in dysfunction and inflammation, and proposed interventions for AEC protection.

Furthermore, the gut–lung axis plays a pivotal role in sepsis immunometabolism. Li et al. elucidated bidirectional regulation: gut dysbiosis impairs barrier function, reduces short-chain fatty acid (SCFA) production, and increases endotoxin translocation, activating alveolar macrophages, neutrophil extracellular traps (NETs), and T-cell imbalances, thereby worsening ALI/ARDS. Core mechanisms involve dynamic interactions between hypoxia-inducible factor-1α (HIF-1α), mammalian target of rapamycin (mTOR), and sirtuins, which regulate immune cell metabolism. Moreover, interventions such as probiotic preparations, fecal microbiota transplantation, and SCFA supplementation can effectively alleviate sepsis-associated lung injury and improve prognosis. By highlighting immunometabolic dysregulation, this research is shifting the treatment strategy from a generalized approach to one of “precision immunometabolic editing.”

The liver, as a metabolic-immune organ, synthesizes acute-phase proteins and cytokines during sepsis. However, excessive inflammatory responses can cause severe hepatocyte damage (sepsis-induced liver injury, SILI). Xu X. et al. systematically described the pathogenesis involving inflammation, oxidative stress, mitochondrial dysfunction, pyroptosis, and autophagy, along with emerging therapeutic strategies. This study provides a theoretical framework and practical reference for future treatment strategies targeting SILI. In addition, the constitutive androstane receptor (CAR), a nuclear receptor primarily expressed in the liver, plays a key role in liver metabolism. Van Dender et al. found that CAR function is inhibited in the liver during sepsis, with hepatocyte nuclear factor 4 α (HNF4α) and peroxisome proliferator-activated receptor α (PPARα) involved in regulating the reduced mRNA transcription level of the Nr1i3 gene encoding the CAR, thereby impairing CAR activity. The above research highlights the role of metabolic dysregulation in sepsis and provides a theoretical basis for metabolic regulation in sepsis.

Reliable biomarkers are essential for early sepsis risk stratification and prognosis. The systemic immune-inflammation index (SII), derived from lymphocyte, neutrophil, and platelet counts, reflects inflammatory and immune status. Xu W. et al. retrospectively analyzed clinical data from 1,015 sepsis patients, using latent class mixed models (LCMM) to identify five SII trajectory subgroups. Among these, Class 1 and Class 4 showed the highest in-hospital mortality risk. Moreover, the SII level within 24 hours of admission showed a U-shaped relationship with in-hospital mortality, meaning both low and high SII levels were associated with higher mortality. In addition, circulating immunoglobulin G (IgG) is an important immune effector molecule. Wang H. et al. first reported the clinical application of the immunoglobulin G N-glycome as a novel potential biomarker for predicting mortality risk in *Escherichia coli*-induced sepsis. This study analyzed serum IgG N-glycome levels in 200 adult sepsis patients and healthy volunteers, comparing the predictive efficacy of the IgG N-glycome with existing risk scores for in-hospital mortality. The results showed that when combined with the Sequential Organ Failure Assessment (SOFA) score, the IgG N-glycome served as a strong predictor of in-hospital mortality.

Pei et al. aimed to identify core genes for the early diagnosis of sepsis. Using three independent transcriptomic datasets of sepsis patients from the Gene Expression Omnibus (GEO) database, they identified 307 highly expressed differentially expressed genes (DEGs) and 72 disease-associated risk genes, ultimately determining SEMA4A, LRPAP1, and NTSR1 as core sepsis-related genes. Using clinical sample single-cell sequencing and *in vitro* experiments, they confirmed the potential key role of SEMA4A in sepsis. Furthermore, Liu et al., based on single-cell multi-omics data analysis, proposed a sepsis “immune clock” model for the first time. This study included 46 relevant studies retrieved from 12 databases. They identified three key phase-defining nodes: monocyte-to-macrophage fate bifurcation at 16–24 h, initiation of TOX-driven CD8+ T-cell exhaustion at 36–48 h, and irreversible immunosuppression beyond 72 h. This research reveals key regulatory nodes from a dynamic, time-series perspective, providing clear time windows and targeting strategies for personalized immune interventions in sepsis.

In summary, this Research Topic emphasizes that immunometabolic dysregulation is a key contributor to the pathophysiology of sepsis and a promising therapeutic target. The articles included in this topic elucidate the molecular mechanisms of immunometabolic dysregulation, validate novel biomarkers, and demonstrate the efficacy of immunometabolism-targeted therapies. By bridging basic and translational research, this topic provides a roadmap for advancing precision medicine strategies aimed at restoring immunometabolic function and improving outcomes in sepsis patients.

